# Evaluation of Bone Mineral Density: Correlating MRI Cervical Vertebral Bone Quality, CT Hounsfield Units, and DEXA T-Scores

**DOI:** 10.3390/medsci13040304

**Published:** 2025-12-04

**Authors:** Rose Fluss, Riana Lo Bu, Alireza Karandish, Sertac Kirnaz, Rafael De la Garza Ramos, Saikiran G. Murthy, Reza Yassari, Yaroslav Gelfand

**Affiliations:** 1 Department of Neurosurgery, Montefiore Medical Center, Bronx, NY 10461, USA; abbani@montefiore.org (R.F.); skirnaz@montefiore.org (S.K.); rdelag@montefiore.org (R.D.l.G.R.); samurthy@montefiore.org (S.G.M.); ryassari@montefiore.org (R.Y.); 2 Dominick P. Purpura Department of Neuroscience, Albert Einstein College of Medicine, Bronx, NY 10461, USA; riana.lobu@einsteinmed.edu (R.L.B.); alireza.karandish@einsteinmed.edu (A.K.)

**Keywords:** bone mineral density (BMD), dual-energy X-ray absorptiometry (DEXA), cervical vertebral bone quality (CVBQ), Hounsfield unit (HU), osteoporosis

## Abstract

Background/Objectives: Dual-energy X-ray absorptiometry (DEXA), the gold standard for assessing bone mineral density (BMD), may yield inaccurate results in certain populations. This has prompted interest in alternative imaging methods, including the MRI-based cervical and lumbar vertebral bone quality (CVBQ and LVBQ) scores. The lumbar VBQ score is a validated MRI-based metric with excellent inter- and intra-rater reliability and established clinical utility in preoperative spine assessment, whereas the newer cervical VBQ (CVBQ) score has shown mixed results in early studies. This study investigates associations between a novel CVBQ score derived from MRI and established BMD metrics (Hounsfield unit (HU) measurements and DEXA values) to evaluate the comparative utility of these methods. Methods: A retrospective review was performed on patients who underwent cervical CT, DEXA, and non-contrast MRI of the cervical and lumbar spine between 2016 and 2022. BMD was assessed using DEXA T-scores, cervical HU measurements, and CVBQ and LVBQ scores derived from T1-weighted MRI sequences. Statistical comparisons between patients with and without osteoporosis were conducted using *t*-tests and Pearson correlation coefficients. Results: A total of 133 patients were included for CVBQ scoring and 127 for LVBQ. The CVBQ score showed poor correlation with both DEXA (r = −0.09, *p* < 0.001) and HU measurements (r = −0.34, *p* < 0.001), whereas a moderate correlation was found between LVBQ and DEXA scores (r = −0.34, *p* < 0.001). Conclusions: The LVBQ score demonstrates moderate correlation with DEXA and may serve as a reliable tool for preoperative BMD assessment. However, the CVBQ score showed weak correlation with both DEXA and HU, limiting its clinical utility in its current form. Further refinement of the CVBQ methodology is needed to enhance its accuracy and relevance for surgical risk assessment and postoperative outcome prediction.

## 1. Introduction

Bone mineral density (BMD) plays a critical role in diagnosing osteoporosis and assessing fracture risk, with dual-energy X-ray absorptiometry (DEXA) being the gold-standard and most widely accepted method in clinical practice. Despite its popularity, DEXA often fails to provide accurate results in certain populations, such as patients with high body mass index (BMI) or preoperative spine patients, in whom BMD assessment is essential for anticipating surgical risks such as hardware failure and subsidence [1,2]. Additionally, DEXA measurements are typically limited to the lumbar spine and hip, potentially missing important regional variations in bone quality, particularly in the cervical spine where instrumentation failure can have devastating neurological consequences [1]. Furthermore, lumbar DEXA measurements may be confounded by degenerative changes, aortic calcification, and previous surgical instrumentation, limiting their reliability in the surgical population [2]. These limitations have created a critical need for alternative, site-specific BMD assessment methods, particularly for patients undergoing cervical spine surgery.

This has spurred interest in alternative imaging methods to evaluate BMD, particularly in the context of spinal fusion candidates. Among these alternatives, the Hounsfield Unit (HU) score, obtained from computed tomography (CT) scans, and the magnetic resonance imaging (MRI)-based lumbar vertebral bone quality (LVBQ) score have garnered attention [3,4,5,6],. CT-based HU measurements have demonstrated strong correlations with BMD and fracture risk, offering the advantage of opportunistic screening from imaging already obtained for surgical planning [3,4]. However, CT imaging involves ionizing radiation and may not be routinely obtained in all surgical candidates, limiting its universal applicability [5]. The LVBQ score, which is derived from MRI signal intensities in the lumbar vertebrae, offers a promising solution for BMD assessment without the drawbacks of DEXA. This technique has shown excellent reliability and can be seamlessly integrated into routine clinical workflows, taking advantage of MRI scans already ordered for preoperative evaluation [6,7]. As a non-invasive, radiation-free alternative, the LVBQ score provides a cost-effective and convenient method for BMD assessment, making it particularly appealing in the context of spine surgery. Since its introduction, the LVBQ score has been validated in multiple studies demonstrating strong correlations with DEXA T-scores and predictive value for complications following lumbar fusion surgery [6,7]. The success of this lumbar-based metric has naturally raised questions about whether similar MRI-based assessments could be developed for the cervical spine.

Consequently, this had led to a recent interest in finding an MRI-based cervical vertebral bone quality (CVBQ) score. Given the anatomical and biomechanical differences between the lumbar and cervical spine, along with distinct MRI signal characteristics in these regions, extrapolating LVBQ findings to the cervical spine requires careful validation. However, the relationship between the CVBQ score and other BMD metrics, such as the HU score and DEXA T-scores, remains controversial with the literature containing few studies with mixed findings [8,9,10],. Specifically, while preliminary studies have suggested potential correlations between CVBQ scores and traditional BMD measures [8,9], other investigations have reported inconsistent results, with correlation coefficients ranging widely and varying levels of clinical utility [10]. These discrepancies may stem from differences in measurement techniques, patient populations, cervical level selection, or MRI sequence parameters. Moreover, no study to date has comprehensively compared CVBQ scores against both CT HU values and DEXA T-scores within the same patient cohort, limiting our understanding of how these modalities relate to one another in the cervical spine specifically. Therefore, there remains a critical gap in understanding whether CVBQ scores can serve as a reliable surrogate for established BMD assessment methods in patients undergoing cervical spine surgery. This study aims to investigate the associations between the CVBQ score and both HU and DEXA values, providing insights into the comparative utility of these different BMD assessment tools in spine surgery patients. Specifically, we hypothesize that CVBQ scores will demonstrate significant correlations with both CT HU values and DEXA T-scores, supporting their potential use as a convenient, radiation-free alternative for BMD assessment in the preoperative evaluation of cervical spine surgery candidates. By establishing these relationships in a well-characterized surgical cohort, this study will help clarify the clinical role of CVBQ scoring and inform evidence-based approaches to preoperative bone quality assessment in cervical spine surgery.

## 2. Materials and Methods

### 2.1. Study Cohorts

A retrospective review was conducted of medical and radiographic records from patients imaged between 2016 and 2022 at a single academic medical center who underwent cervical CT on a nonquantitative General Electric scanner, DEXA, as well as synchronous MRI without contrast of the cervical and lumbar spine, with all imaging modalities performed within a 24-month interval for each patient. The 24-month timeframe was selected to minimize temporal variability in bone quality while maximizing sample size, as bone density changes typically occur gradually over years in the absence of major metabolic events or therapeutic interventions. Eligible cases were identified through a systematic review of the Picture Archiving and Communication System (PACS), beginning in January 2016, to locate patients meeting these imaging criteria. This systematic approach was chosen to ensure consecutive patient enrollment and minimize selection bias. The requirement for all three imaging modalities (CT, MRI, and DEXA) within the specified timeframe was based on the study’s primary objective of comparing BMD assessment methods within the same patient cohort, as cross-sectional comparison provides the most accurate assessment of inter-modality correlations.

Inclusion criteria were: (1) age ≥ 18 years; (2) availability of cervical CT, DEXA, and non-contrast MRI of both the cervical and lumbar spine obtained within a 24-month period; and (3) imaging performed on a General Electric CT scanner.

Exclusion criteria were: (1) poor-quality MRI due to motion artifacts; (2) absence of T1-weighted sequences; (3) prior spinal instrumentation or fracture involving the region of interest (C3–C6 or L1–L4) (as hardware artifacts and post-fracture remodeling can significantly alter signal intensity and HU values, compromising measurement accuracy); or (4) incomplete imaging or missing records within the study window. No BMI-based exclusions, nor any additional exclusion criteria, were applied.

This study was conducted retrospectively using existing imaging data and patient records. Institutional Review Board (IRB) approval was waived in accordance with institutional policies governing retrospective studies. All patients had previously provided consent for their clinical imaging procedures. All data were de-identified prior to analysis in compliance with HIPAA regulations.

### 2.2. Imaging Protocol

All DEXA scans (lumbar, femoral, and wrist) were obtained using Hologic scanners (Horizon A and Horizon C). APEX software (version 5.6.05) was used to automatically calculate T- and Z-scores. All CT scans were obtained using a General Electric Lightspeed VCT 64 Slice CT Scanner (Waukesha, WI, USA). The HU measurement for each vertebra was obtained by two independent reviewers using PACS software(version 7.4). Reviewers were blinded to DEXA and VBQ results to prevent measurement bias. Each HU measurement was acquired by drawing an elliptical ROI at the midsagittal body excluding the cortical margin. The ROI was standardized to encompass approximately 50–70% of the vertebral body cross-sectional area, positioned centrally to avoid cortical bone, endplates, and posterior elements, which could artificially elevate HU values. CT parameters included a slice thickness of 0.625 mm with 0.625 mm intervals and imaging from C4 to C6 and L1 to L4. Cervical and lumbar VBQ scores were calculated from the same Philips Ingenia 3T MRI (New York, NY, USA). Scanning parameters were fractional anisotropy (FA) 90.00, TR 1250 msec, TE 80 msec, and slice thickness 4.5 mm.

**Figure 1:**
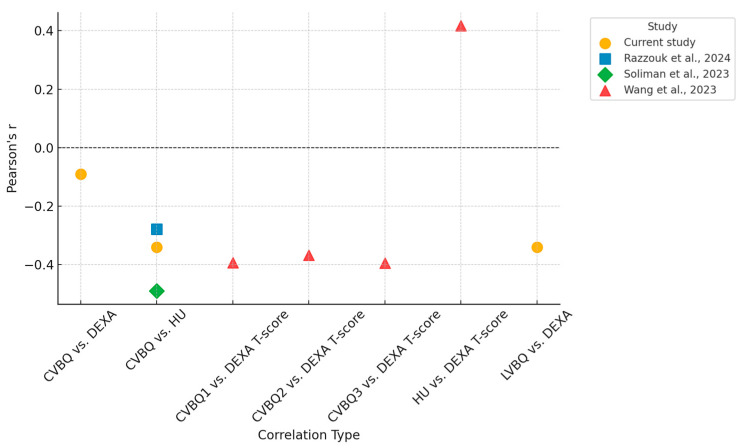
Comparison of Correlations of different BMD assessments across the available literature [8,9,10].

**Figure 2:**
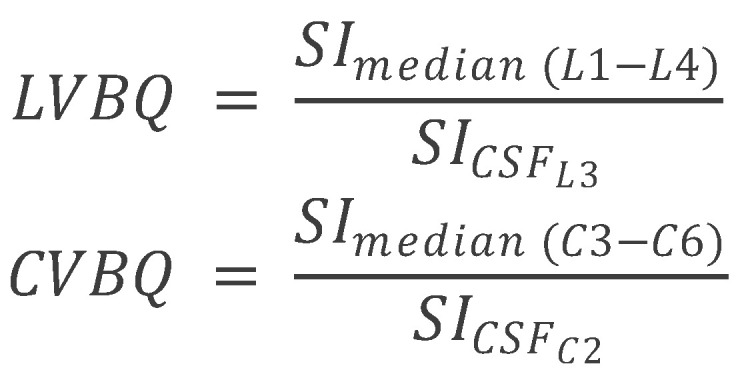
Methodology used for the calculation of LVBQ & CVBQ in the current investigation.

**Figure 3:**
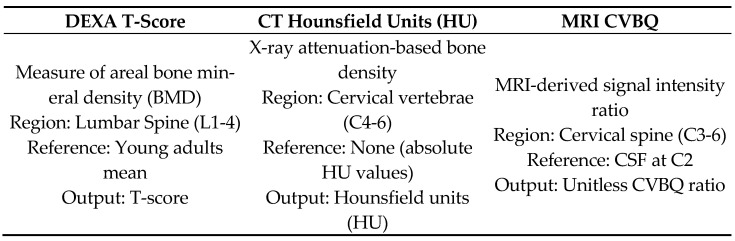
Conceptual schematic comparing DEXA T-scores (areal BMD), CT Hounsfield Units (HU), and MRI-derived CVBQ (signal-intensity ratio). The diagram highlights the typical anatomical regions sampled (lumbar L1–L4 for DEXA; cervical C4–C6 for HU and CVBQ), the reference tissue used for CVBQ normalization (CSF at C2), and each method’s output scale (T-score, HU, unitless ratio).

The lumbar vertebral bone quality (VBQ) score was calculated from non-contrast, T1-weighted MRI sequences of the cervical, and lumbar spine. The VBQ score is based on the principle that T1-weighted signal intensity of vertebral bone marrow reflects its composition: higher fat content (seen in osteoporotic bone with yellow marrow replacement of hematopoietic red marrow) produces higher T1 signal, while lower signal indicates preserved bone quality. By normalizing vertebral signal intensity to CSF (which has consistent signal characteristics), the VBQ score provides a quantitative, scanner-independent measure of bone quality [6,7]. Mid-sagittal T2-weighted MRI sequences were used to identify cerebrospinal fluid (CSF) regions for reference. T2-weighted images provide superior CSF visualization due to the long T2 relaxation time of CSF, facilitating accurate ROI placement. The VBQ score for the lumbar spine was determined as described by Ehresman et al., using regions of interest (ROI) placed within the medullary portions of the L1–L4 vertebral bodies and CSF at the L3 level [11]. ROIs were circular with a standardized diameter of 10 mm, positioned in the center of each vertebral body on mid-sagittal T1-weighted images to avoid cortical bone, basivertebral veins, and Schmorl’s nodes, which could confound measurements. The L3 CSF ROI was positioned in the mid-thecal sac, avoiding nerve roots and flow artifacts. The median signal intensity (MSI) of the L1–L4 vertebral bodies was divided by the signal intensity of CSF at L3 to calculate the VBQ score. Similar procedures were followed for the cervical regions, with VBQ scores derived from C3–C6 vertebrae and C2 CSF as described by Soliman et al. [8]. Unlike Wang et al., who reported CVBQ1–3 variants to reflect ROI selection at different levels [9], we prespecified a single, unified CVBQ derived from the mean of C3–C6 normalized to CSF at C2 to prioritize cross-scanner reproducibility and analytic simplicity (Figure 1 and Figure 2). To illustrate the relationship between these imaging-based bone-quality methods, we created a schematic overview comparing DEXA T-scores, CT HU, and MRI-derived CVBQ (Figure 3).

### 2.3. Statistical Analysis

For statistical analysis, data were collected and visualized using Microsoft Excel (version 16.0, 2016). Statistical significance was defined as *p* < 0.05 for all analyses. Assumptions for parametric testing were verified with Levene’s test for homogeneity of variances prior to applying *t*-tests and ANOVA.

Categorical variables are presented as numbers and continuous variables as means and confidence intervals. Pearson’s correlation coefficient was used to correlate (1) lumbar VBQ to DEXA, (2) CVBQ and DEXA, (3) CVBQ and HU. Correlation coefficients were categorized as weak (0–0.3), moderate (0.3–0.7), or strong (0.7–1.0). One-way analysis of variance (ANOVA) was performed to assess differences among cervical VBQ scores for osteoporotic, osteopenic, and normal patients and *t*-test was performed to assess differences among cervical VBQ scores for osteoporotic and non-osteoporotic patients. Consistent with statistical equivalence of two-group ANOVA and the independent-samples *t*-test, we used *t*-tests whenever only two categories were compared.

Similarly to prior studies, the classification of DEXA bone density categories was based on the lumbar T-score according to the WHO criteria (normal BMD: T-score of −1 or higher, osteopenia: T-score between −1 and −2.5, osteoporosis: T-score of −2.5 or lower). An additional classification label was defined based on cervical HU measurements according to values established by Fluss et al. (normal BMD: HU measurement of 340.98 or higher, osteopenia: HU measurement between 326.5 and 340.98, osteoporosis: HU measurement of 326.5 or lower) [5]. A *t*-test was performed in this case instead of an ANOVA as the classification by HU identified only 1 patient as having osteopenia, leading to invalid results using 3 categories. Therefore, the osteopenia patient in the sample was grouped with the patients labeled Normal and the *t*-test was used to compare patients with and without osteoporosis. A direct correlation between DEXA and HU was not computed because DEXA values were obtained at lumbar sites whereas HU values were measured in the cervical spine, making cross-regional comparisons methodologically inappropriate.

To visualize distributional patterns beyond correlation coefficients, scatterplots with fitted simple linear regression lines were generated for DEXA vs. CVBQ and CVBQ vs. HU. These plots were produced using the subset of patients with complete DEXA, CT, and MRI data (*n* = 34).

## 3. Results

A total of 133 patients were included for calculation of cervical vertebral bone quality (CVBQ) scores and 124 patients for lumbar vertebral bone quality (LVBQ) scores. Among these, 85 patients underwent both lumbar CT and DEXA, and 128 patients underwent both cervical and lumbar CT imaging. Within this cohort, 34 patients had cervical MRI, cervical CT, and DEXA, while 33 patients had lumbar MRI, lumbar CT, and DEXA.

The cohort used for CVBQ analysis (n = 133) had a mean age of 61.9 years (median 63), and the LVBQ cohort (*n* = 124) had a mean age of 65.6 years (median 67). Among the 34 patients with cervical MRI, CT, and DEXA, 3 were male and 31 were female, while the 33 patients with lumbar MRI, CT, and DEXA included 5 males and 28 females (Table 1).

The mean VBQ scores were 2.803 for the cervical region and 2.852 for the lumbar region. A weak correlation was observed between CVBQ and DEXA scores (Pearson r = −0.09, *p* < 0.001), whereas a moderate correlation was found between LVBQ and DEXA scores (r = −0.34, *p* < 0.001). Similarly, a moderate correlation was observed between CVBQ and Hounsfield Unit (HU) measurements (r = −0.34, *p* < 0.001).

**Figure 4:**
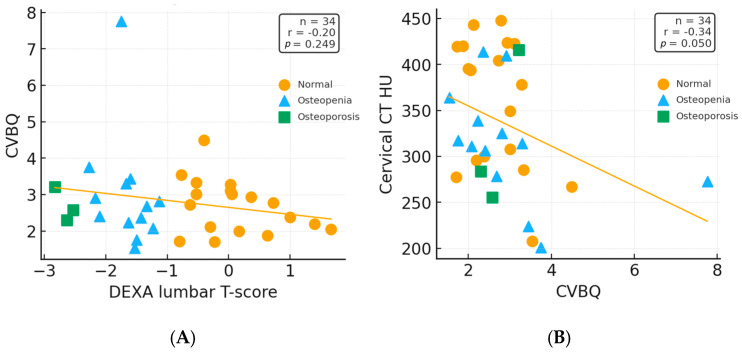
(**A**) DEXA lumbar T-score vs. cervical vertebral bone quality (CVBQ) and (**B**) CVBQ vs. cervical CT Hounsfield Units (HU), each with fitted linear regression lines. Marker shape and color denote WHO bone-density category based on lumbar T-score. Axes were assigned to reflect each modality’s reference role: DEXA values (the clinical standard) are plotted on the *x*-axis in (**A**), while CT HU values (the radiologic comparator) are plotted on the *y*-axis in (**B**). While these plots illustrate the general direction of association, full-cohort correlations are reported in Table 2 (r = −0.09 for CVBQ–DEXA and r = −0.34 for CVBQ–HU, both *p* < 0.001).

Table 2 summarizes these Pearson correlation coefficients alongside published values for comparison. Figure 4 illustrates these relationships in the subset of patients (n = 34) who underwent all three imaging modalities (DEXA, CT, and MRI). Fitted linear regression lines demonstrate weak to moderate linear trends consistent with those observed in the full cohort.

A *t*-test comparing CVBQ scores between HU-defined categories for osteopenia (mean HU = 340.98) and osteoporosis (mean HU = 326.5) yielded *p* = 0.119, indicating no statistically significant difference in CVBQ across these diagnostic groups.

## 4. Discussion

The lumbar VBQ score, an MRI-based tool for assessing BMD, has been a validated metric against DEXA and is regarded for its excellent interrater and inter-rater reliability [6,7]. Among various MRI-based methods for BMD evaluation, the lumbar VBQ score has emerged as an acceptable and practical clinically applicable tool, particularly for preoperative spine patients [12]. The recently developed cervical CVBQ score has shown mixed results in the literature, with our study revealing a weak correlation between CVBQ and DEXA scores [9,10,13,14,15,16]. Consistent with existing studies, we found a moderate correlation between lumbar VBQ and DEXA scores, as well as a moderate association between cervical VBQ and Hounsfield Unit (HU) measurements [10].

An important finding in our analysis is the patient with markedly elevated CVBQ (>7.5) and correspondingly low HU (~175), yet near-average DEXA measurements. This discordance exemplifies a well-recognized limitation of DEXA: bone mineral density can be confounded by degenerative changes, vertebral compression, osteophytes, and vascular calcifications that overlay the spine. These structural abnormalities artificially elevate DEXA T-scores while having minimal impact on volumetric trabecular density measurements (HU) obtained from defined regions of interest within vertebral bodies, or on morphometric fracture assessment (CVBQ). We retained this patient in our primary analysis because the findings represent a clinically meaningful scenario, which underscores the value of CT-based bone assessment in patients with advanced osteoporotic disease where DEXA may be unreliable. However, the paucity of patients with severe vertebral deformity in our cohort limits definitive conclusions about the CVBQ-HU relationship at extreme values, warranting validation in larger studies with broader disease severity representation.

DEXA has served as the clinical gold standard since the late 20th century while CT-based Hounsfield units have been leveraged as a surrogate for bone quality for over a decade in spine populations [17,18,19]. The large appeal of the VBQ score is its ability to take advantage of existing MRI data, which is routinely ordered for spine patients. Unlike CT-based methods such as HU, which often require additional imaging and may be subject to insurance constraints, VBQ scoring can be performed opportunistically using pre-existing scans, without incurring additional radiation, cost, or time. This makes VBQ a highly practical tool for clinical use, especially in cases where DEXA scans are either not available or not ordered, and where these advanced imaging modalities are cost-prohibitive or less readily accessible. The ease of implementation and rapid results, unaffected by factors such as patient weight or body composition, position VBQ as an attractive alternative to traditional BMD measurement techniques [1].

Despite its potential, further optimization and validation of the VBQ score are essential to ensure its broader applicability across various clinical scenarios. The moderate correlation observed between LVBQ and DEXA further supports the idea that these two regions may offer comparable insights into overall spinal BMD and recent investigations on its predictive value in subsidence has strengthened the clinical relevance of these associations [20]. Given that cervical VBQ scores do not robustly align with DEXA, or HU measurements it would be premature to use cervical VBQ as a substitute for DEXA in determining clinical decision-making related to osteoporosis or fracture risk in regard to the cervical spine. However, cervical VBQ has shown utility in evaluating BMD for predicting post-operative cage subsidence in the cervical spine [21]. While the cervical VBQ could provide some predictive value for bone quality in the setting of surgical hardware it should not be directly substituted for DEXA or cervical HU in clinical practice as this investigation is the second one of its nature to refute its diagnostic validity as a stand-alone measure.

The aim of this current study was to potentially establish clear thresholds for CVBQ scores, similar to those already established for cervical HU, to provide clinicians with reliable cut-off values for diagnosing osteoporosis and osteopenia. However, this investigation failed to validate CVBQ scores against quantitative CT and other gold-standard BMD measures which questions its role as a diagnostic tool. This study very closely supports the conclusions made by Razzouk et al. with nearly identical Pearson correlation coefficients found between our investigations [10]. (Table 2) Perhaps CVBQ should not be viewed merely as a surrogate for BMD as defined by DEXA or cervical HU; instead, these measurements should be considered as parallel tools, each offering distinctive insights in the management of cervical spine-related conditions, namely preoperative risk stratification versus long-term monitoring of bone health.

Poor bone quality has long been recognized as a significant contributor to complications following spinal fusion, including issues like proximal junctional kyphosis, hardware failure, and the potential need for reoperation [22,23]. While dual-energy X-ray absorptiometry (DEXA) remains the gold standard for BMD assessment, it is not always the most accurate or suitable option, particularly in certain patient populations or clinical scenarios [2]. In such cases, MRI-based assessments like the VBQ score are tailored to specific regions of the spine which may offer a more flexible and precise alternative, better aligned with the anatomical focus of the patient’s condition. Thus, the findings of this investigation do not necessarily conclude that CVBQ should not be used given its poor correlation with DEXA, but rather that CVBQ may be measuring an aspect of BMD more useful in the preoperative assessment of surgical patients than DEXA.

Huang et al. reported a significant correlation between a cervical MRI-based bone quality score and DEXA T-scores, with an overall accuracy of 0.78 [24]. The discrepancy between these findings and our own in addition to Razzouk et al. could potentially be attributed to variance of MRI machine used [24,25]. Although VBQ scores are generally felt to be independent of the specific MRI machine since the analysis relies on a standardized reference point within the image, typically the cerebrospinal fluid signal intensity, which facilitates normalization across various machines [24,25]. However, factors such as magnetic field strength and bandwidth may still have a minor influence on the scores, potentially contributing to variations in findings. Notably, among the available literature, both studies conducted in North America (which includes this current investigation) found no significant correlation between CVBQ and either HU or DEXA. In contrast, two well-conducted studies of similar methodology in China demonstrated strong correlations with DEXA T-scores [8,9,10]. A comparison of these studies is provided in Table 2.

Another factor that may explain the difference in results is the methodology used to calculate cervical VBQ scores. We followed the approach established by Ehresman et al. for the lumbar spine and Soliman et al. for the cervical spine [8,11]. The methods used in the current article involved placing regions of interest (ROIs) within the C3–C6 vertebral bodies and the CSF at the level of C2. The median signal intensity (MSI) from C3–C6 was then calculated, and the VBQ score was derived as the quotient of MSI from C3–C6 divided by the SI of CSF at C2. However slight variations in methodologies used in comparative investigations exists and this methodological difference in how cervical VBQ scores were calculated may explain the divergent conclusions between the studies.

Future studies can explore whether a more refined calculation for cervical regions, potentially expanding the analysis to include the entire cervical (C1–C7) which could improve the accuracy and reliability of these measures. Optimizing these methods could lead to more robust and clinically relevant BMD assessments across different spinal regions. Future work may consider innovations in the MRI-based calculation of VBQ scores to further improve their utility.

Our study has several limitations that must be considered when interpreting these results. First, although our results were nearly identical to those of Razzouk et al., the sample size in this study may have been insufficient to detect an accurate correlation between CVBQ, HU and DEXA [10]. Larger, more diverse populations could provide greater statistical power and perhaps uncover relevant associations that were not observed here. Additionally, the single-institution design may limit generalizability, though it also ensures consistent imaging protocols and eliminates inter-institutional variability in acquisition and post-processing. We focused on VBQ because it is the most validated MRI-derived metric in operative spine cohorts; alternative MRI or CT methods (e.g., texture analysis or QCT) were not pursued given limited standardization and our sample size, which would have precluded robust comparative statistics.

## 5. Conclusions

The cervical CVBQ score demonstrated a weak correlation to DEXA and Hounsfield Unit (HU) measurements in our study. This suggests that CVBQ may not yet be a reliable substitute for DEXA or HU in determining osteoporosis or fracture risk in the cervical spine, although it may still hold value in predicting postoperative outcomes, such as cage subsidence. Despite these findings, the ability of VBQ to leverage existing MRI data, avoiding the need for additional imaging or radiation, positions it as an attractive tool for clinicians. While cervical VBQ’s current role in clinical practice remains limited, future studies will continue to explore how it can serve as a valuable adjunct in certain situations, particularly for surgical risk assessment.

## Figures and Tables

**Table 1:** Demographic Characteristics of CVBQ and LVBQ Cohorts.

	Cervical MRI Cohort (n = 133)	Lumbar MRI Cohort (n = 127)
Mean Age (years)	61.9	65.6
Median Age (years)	63	67
Demographic Characteristics of Combined Imaging Subgroups	Cervical MRI + CT + DEXA (n = 34)	Lumbar MRI + CT + DEXA (n = 33)
Gender (M/F)	3/31	5/28

**Table 2:** Pearson’s Correlation Coefficients (r) Between MRI-Based VBQ Scores and Standard BMD Assessment Methods (DEXA and HU) Across Studies.

Study	CVBQ vs. DEXA	CVBQ vs. HU	CVBQ1 vs. DEXA T-Score	CVBQ2 vs. DEXA T-Score	CVBQ3 vs. DEXA T-Score	HU vs. DEXA T-Score	LVBQ vs. DEXA
Current study	−0.09 **	−0.34 **					−0.34 **
Razzouk et al., 2024 [10]		−0.279 *					
Soliman et al., 2023 [8]		−0.49 **					
Wang et al., 2023 [9]			−0.393 **	−0.368 **	−0.395 **	0.417 **	

* Statistically significant (*p* = 0.015). ** Highly significant (*p* < 0.001).

## Data Availability

The data presented in this study are available on request from the corresponding author. The data are not publicly available due to privacy and ethical restrictions.

## Abbreviations

The following abbreviations are used in this manuscript:

BMD	Bone mineral density
DEXA	Dual-energy X-ray absorptiometry
HU	Hounsfield Unit
MRI	Magnetic resonance imaging
VBQ	Vertebral Bone Quality (MRI-based)
LVBQ	Lumbar Vertebral Bone Quality
CVBQ	Cervical Vertebral Bone Quality
CVBQ1-3	CVBQ variants reported at distinct cervical levels (per Wang et al.)
CSF	Cerebrospinal fluid
ROI	Region of interest
ANOVA	Analysis of variance
BMI	Body Mass Index
CT	Computed Tomography
PACS	Picture Archiving and Communication System
IRB	Institutional Review Board
MSI	Median Signal Intensity
